# Food groups intake of cirrhotic patients, comparison with the nutritional status and disease stage 

**Published:** 2019

**Authors:** Fereshteh Pashayee-khamene, Mahdi Saber-firoozi, Behzad Hatami, Bahram Rashidkhani, Vahideh Aghamohammadi, Elahe Mohammadi, Azita Hekmatdoost

**Affiliations:** 1 *Department of Clinical Nutrition and Dietetics, Faculty of Nutrition Sciences and Food Technology, National Nutrition and Food Technology Research Institute, Shahid Beheshti University of Medical Sciences, Tehran, Iran*; 2 *Digestive Disease Research Center, Digestive Disease Research Institute, Tehran University of Medical Science, Tehran, Iran*; 3 *Gastroenterology and Liver Diseases Research Center, Research Institute for Gastroenterology and Liver Diseases, Shahid Beheshti University of Medical Sciences, Tehran, Iran*; 4 *Department of Community Nutrition, Faculty of Nutrition Sciences and Food Technology, National Nutrition and Food Technology Research Institute, Shahid Beheshti University of Medical Sciences, Tehran, Iran*; 5 *Department of Paramedical Sciences, Ahvaz Jundishapur University of Medical Sciences, Ahvaz, Iran*; 6 *Faculty of Nutrition, Khalkhal University of Medical Sciences, Khalkhal, Iran*

**Keywords:** food groups, hepatic cirrhosis, malnutrition, Child-Pugh score

## Abstract

**Aim:**

The aim of this study was to determine the relation between different food groups intake, nutritional status of cirrhotic patient and the stage of the disease.

**Background:**

Protein-energy malnutrition (PEM) is a common problem in cirrhotic patients. Food intake assessment is highly important in the investigation regarding the health-disease process.

**Methods:**

In this cross-sectional study, sixty eight ambulatory cirrhotic patients, with a mean age of 54 years, were included. In order to assess the stage of the disease and malnutrition status, Child-Pugh score and Subjective Global Assessment index were used respectively. Dietary intakes were assessed using a 168-item semi-quantitative validated food frequency questionnaire. Odds ratios (OR) and the corresponding 95% confidence intervals (CI) were computed, using logistic regression models.

**Results:**

After adjustment for confounders, we found significant inverse relations between intakes of nuts (OR=0.140, CI=0.031-0.625) and olive (OR=0.212, CI=0.049-0.917) with severity of disease and boiled potatoes (OR=0.154, CI=0.040-0.592) and legumes (OR=0.090, CI=0.020-0.406) with malnutrition status. Inversely, solid fats (OR=3.324, CI=1.080-10.238) and mayonnaise (OR=5.215, CI=1.203-22.612) were positively associated with disease severity and malnutrition, respectively.

**Conclusion:**

These findings suggest that selection of healthy foods was negatively associated with severity of hepatic cirrhosis whereas unhealthy food groups had a positive relation with disease severity and malnutrition.

## Introduction

 Protein energy malnutrition (PEM) is highly prevalent in cirrhotic patients and differs from 60-100% in decompensated to 20-30% in compensated patients and even reaches to 75% in ambulatory patients (1). Patients with severe disease (Child-Pugh’s grading of B and C) usually suffer from severe malnutrition (1). PEM would adversely affect the general and clinical status, liver function, morbidity and mortality in cirrhotic patients as well as reduced survival when such patients undergo liver transplantation (2, 3). 

Several factors contribute to malnutrition including poor oral intake, malabsorption, metabolic abnormality, increased energy requirement and disturbances in substrate utilization such as reduced glucose oxidation and increased lipid oxidation, accelerated protein breakdown and inefficient patient’s synthesis (4). 

Assessment of dietary intake is crucial for monitoring the disease course in cirrhotic patients. There are several methods to evaluate the quality and quantity of nutrient intake in liver disease patients; however, Subjective Global Assessment (SGA) is considered an optimal method because it does not include the parameters of nutritional status or biochemical values which might be affected by liver dysfunction (2, 5). It has been shown that the progression of malnutrition is associated with disease severity (6). Generally accepted methods for assessing the clinical status and severity of disease in cirrhotic patients are the Child-Pugh-Turcotte classification (5, 7). Although some studies have shown the high prevalence of malnutrition in cirrhotic patients and its relation with disease prognosis, there is no study evaluating the role of various food items in disease progression and malnutrition. Therefore, assessing dietary behavior of cirrhotic patients appears essential to increase the knowledge in this domain and to identify the role of nutritional factors in disease progression.

The present cross-sectional study aimed to evaluate the association between dietary food groups, nutritional status of the patient and the stage of the disease among Iranian populations. 

## Methods

**Subjects**

In this cross-sectional study, sixty eight ambulatory cirrhotic patients (with more than 6 months of cirrhosis diagnosis), with a mean age of 54 years, were recruited from two educational hospitals of Tehran (capital of Iran) from September 2016 to February 2017. Majority of patients (67.7%) had hepatitis C viral etiology and were male (72%). All patients gave written informed consent before inclusion (participation rate >97%). Exclusion criteria were the following: pregnancy, chronic renal or cardiac diseases, diabetes mellitus, pancreatic insufficiency, neoplasia and acquired immuno deficiency syndrome. The diagnosis of cirrhosis was based on the medical history, physical examination, biochemical findings and imagistic methods (ultrasound and /or computed tomography). Study protocol was approved by the Ethical Committee for Research at Shahid Beheshti University of Medical Sciences with ethical code Ir.sbmu.nnftri.1396.186.

**Dietary assessment**

We evaluated dietary intake of patients using a validated Food Frequency questionnaire (FFQ) (8). The questionnaire included 168 food and beverage items, categorized in 13 food groups (vegetables, fruits, dairy, grains, animal source proteins, liquid oil, sweets, boiled potato, nuts, solid fats, legumes, mayonnaise, and olive). Subjects were asked to specify their frequency of consumption for each food item on a daily, weekly, monthly or yearly basis. Intakes were then converted to daily frequencies and a manual for household measures was used to convert intake frequencies to grams of food intake/day. For each group portion size was estimated using either usual containers (for example spoon or standard unit as yogurt) or a set of validated color photographs. 

**Nutritional assessment**

Nutritional assessment was accomplished by using SGA according to the proposition of Destky *et al*. (9). The standard SGA includes nutritional evaluation of height, weight (current, before illness and weight variation in the previous 6 months), nutritional history (appetite, intake, and gastrointestinal symptoms), physical examination assessment of fat loss, muscle wasting, and presence of ascites or encephalopathy, infections and renal insufficiency. Body weight was measured in lightweight clothing and without shoes to the nearest 0.5 kg, using a scale (Seca, Germany). Height was measured by a mounted tape without shoes to the nearest 0.1 cm accuracy using a stadiometer (Seca, Germany). Body mass index (BMI) was calculated as the weight in kilogram divided by the square of the height in meters. Based on this evaluation, patients were classified prospectively into three groups: A: well-nourished B: moderately malnourished C: severely malnourished. 

The severity of liver disease was assessed by the child-pugh classification. The score was calculated by serum albumin, total bilirubin, international normalized ratio (INR), and presence of ascites or encephalopathy.

**Covariates**

Data about lifestyle (educational level, socioeconomic status, marital status, smoking status, and physical activity level), nutritional behaviors (alcohol consumption and food supplements) and medical history (such as high blood pressure, diabetes) and current treatment were collected at inclusion using questionnaires. Anthropometric indices were measured accurately (10).

**Statistical analysis**

We performed statistical analyzes of the data by using SPSS (Statistical Package for the Social Science) version.22. A p value of <0.05 was taken as significant. Due to the low number of patients in C class of child-pugh and SGA, they were stratified into two groups (conversion of 3-state to 2-state). The quantitative variables were expressed as mean and standard deviation, and qualitative variables were shown by absolute and relative frequencies. To compare means in two groups, the Student t test was used. The chi-square test of Pearson was applied for categorical variables. We used logistic regression models to examine the association between different food group consumption, severity of malnutrition and stage of the disease. Logistic regression was adjusted for age and energy in model 1; and age, energy, BMI, smoking and alcohol consumption in model 2.

**Table 1:** Characteristics of participants (n=68) according to Child-pugh score

**Characteristics**	**Total participants (n =68)**	**Child-pugh**	
		**A (n=47)**	**B and C(n=21)**
	**Mean±SD**	**Mean±SD**	**Mean±SD**
Age	54.63±11.67	54.70±11.81	54.47±11.64
Weight	73.69±15.10	76.36±16.19	68.59±10.38^b^
Height	167.38±7.78	169.14±7.54	163.42±6.96^b^
BMI	26.68±4.99	27.00±5.50	25.95±3.63
	**n (%)**	**n (%)**	**n (%)**
Etiology			
Virus	44(67.7)	32(72.7)	12(27.3)
other	21(32.3)	14(66.7)	7(33.3)
Sex			
Male	49(72.1)	34(69.4)	15(30.6)
female	19(27.9)	13(68.4)	6(31.6)
Obesity			
Yes	21(30.9)	17(81)	4(19)
No	47(69.1)	30(63.8)	17(36.2)
Smoking			
Yes	30(46.2)	22(73.3)	8(26.7)
No	35(53.8)	23(65.7)	12(34.3)
Alcohol consumption			
Yes	18(28.1)	14(77.8)	4(22.2)
No	46(71.9)	30(65.2)	16(34.8)
Calcium supplementation			
Yes	5(7.4)	5(100)	0(0)
No	63(92.6)	42(66.7)	21(33.3)
multivitamin supplementation			
Yes	27(39.7)	18(66.7)	9(33.3)
No	41(60.3)	29(70.7)	12(29.3)

BMI: body mass index;

a T-test or Mann-Whitney U test were used for comparison of quantitative variables and chi-square test or Fisher’s exact test were used for comparison of qualitative variables;

b p value< 0.05

**Table 2:** Association of food groups intake (according to median) with Child-Pugh score

P value*	**Severity of hepatic insufficiency**		**Intake (g/day) **	**Food group**
	**Child-pugh B and C** **n (%)**	**Child-pugh A** **n (%)**		
0.018^a^	15(71.4)	19(40.4)	Low	Nuts
	6(28.6)	28(59.6)	High	
0.023^a^	18(85.7)	27(57.4)	Low	Olive
	3(14.3)	20(42.6)	High	
0.030^a^	7(33.3)	29(61.7)	Low	Solid fats
	14(66.7)	18(38.3)	High	
0.793	11(52.4)	23(48.9)	Low	Vegetables
	10(47.6)	24(51.1)	High	
0.431	12(57.1)	22(46.8)	Low	Fruits
	9(42.9)	25(53.2)	High	
0.431	12(57.1)	22(46.8)	Low	Dairy
	9(42.9)	25(53.2)	High	
1.000	10(47.6)	24(51.1)	low	Grains
	11(52.4)	23(48.9)	high	
1.000	11(52.4)	23(48.9)	low	Animal protein
	10(47.6)	24(51.1)	high	
0.294	13(61.9)	21(44.7)	low	Liquid oil
	8(38.1)	26(55.3)	high	
0.600	9(42.9)	25(53.2)	low	Sweets
	12(57.1)	22(46.8)	high	

a p<0.05; Low and high is according to median; Fisher exact test or chi-square test; n (%) = Number and percent of patients

**Table 3:** Association of different food groups intake (according to median) with severity of malnutrition

**Food groups**	**Intake (g/day)**	**Severity of malnutrition**			**P value** ^a^
		**SGA A** **n (%)**	**SGA B** **n (%)**	**SGA C** **n (%)**	
Boiled potato	Low	9(33.3)	23(69.7)	3(37.5)	0.019^a^
	High	18(66.7)	10(30.3)	5(62.5)	
Legume	Low	7(25.9)	21(63.6)	6(75)	0.008^a^
	High	20(74.1)	12(36.4)	2(25)	
Mayonnaise	Low	21(77.8)	18(54.5)	8(100)	0.020^a^
	High	6(22.2)	15(45.5)	0(0)	
Vegetable	Low	10(37)	18(54.5)	6(75)	0.137
	High	17(63)	15(45.5)	2(25)	
Fruit	Low	11(40.7)	17(51.5)	6(75)	0.238
	High	16(59.3)	16(48.5)	2(25)	
Dairy	Low	13(48.1)	16(48.5)	5 (62.5)	0.885
	High	14(51.9)	17(51.5)	3(37.5)	
Grains	Low	12(44.4)	17(51.5)	5(62.5)	0.622
	High	15(55.6)	16(48.5)	3(37.5)	
Animal protein	Low	13(48.1)	15(45.5)	6(75)	0.380
	High	14(51.9)	18(54.5)	2(25)	
Liquid oil	Low	10(37)	19(57.6)	5(62.5)	0.208
	High	17(63)	14(42.4)	3(37.5)	
Sweets	Low	13(48.1)	17(51.5)	4(50)	1.000

**Table 4:** Odds ratio (OR) and 95% (CI) of malnutrition and severity of disease by food groups intake according to adjusted model

**Child pugh-category**	**SGA-catergory **		**Food Group(g/day)**
0.396(0.134-1.170)	0.260 (0.088-0.766)*	Model 1	Boiled potato (12.1 vs. <12.1≤)
0.408(0.132-1.257)	0.154(0.040-0.592)*	Model 2	
0.583(0.195-1.749)	0.210(0.069-0.637)*	Model 1	Legume (59.25 vs. <59.25≤)
0.411(0.119-1.418)	0.090(0.020-0.406)*	Model 2	
2.157(0.709-6.562)	3.119(1.01-11.000)*	Model 1	Mayonnaise (0 vs. <0≤)
2.049(0.591-7.103)	5.215(1.203-22.612)*	Model 2	
0.190(0.054-0.666)*	0.525(0.180-1.537)	Model 1	Nut (11.3 vs. <11.3≤)
0.140(0.031-0.625)*	0.324(0.090-1.168)	Model 2	
0.208(0.052-0.828)*	0.869(0.302-2.500)	Model 1	Olive (0 vs. <0≤)
0.212(0.049-0.917)*	0.909(0.276-2.996)	Model 2	
3.324(1.080-10.238)*	4.488(1.353-14.886)	Model 1	Solid Fat (2 vs. <2≤)
3.326(0.980-11.291)	4.734(1.203-18.635)	Model 2	
1.030(0.314-3.82)	1.189(0.372-3.799)	Model 1	Grains )≥399.3 vs.<399.3)
0.95(0.250-3.275)	1.130(0.307-4.161)	Model 2	
0.800(0.269-2.383)	1.154(0.402-3.312)	Model 1	Animal protein source )≥123.75 vs.<123.75)
0.913(0.266-3.128)	0.993(0.283-3.485)	Model2	
0.396(0.124-1.263)	0.538(0.184-1.569)	Model 1	Liquid oil )≥12.3 vs.<12.3)
0.415(0.125-1.385)	0.570(0.171-1.904)	Model 2	
1.470(0.485-4.455)	1.307(0.443-3.852)	Model 1	Sweets )≥49.75 vs.<49.75)
2.005(0.599-6.715)	1.038(0.312-3.450)	Model 2	

* p<0.05; Model 1: adjusted for energy and age; Model 2: model 1 plus BMI, smoking and alcohol consumption

## Results

Table 1 shows the demographic characterization according to the severity of cirrhosis. This study included 68 adult cirrhotic patients with a mean age of 54.63years. Patients were mainly men (72.1%) and the most prevalent etiology of hepatic cirrhosis was hepatitis C virus (67.7%). As for the severity of the disease, the majority of patients, 47 (69.1%), were Child-Pugh A, while 21 (30.9%) were Child-Pugh B and only one patient was assigned to Child-Pugh C. Child-Pugh A patients were significantly different from Child-Pugh B and C patients in the case of weight and height (p≤0.05). 

We categorized the amount of food groups intake (gr/day) to low and high levels according to median; then we compared the distributions of these levels of intake across the categories of Child-Pugh in Table 2 and malnutrition status in Table 3. There were significant relationship between nuts, olive and solid fat groups with Child-Pugh score (p≤0.05) (Table 2). Moreover, boiled potatoes, legumes and mayonnaise had statistically significant association with malnutrition classification (Table 3). 

Table 4 shows the odd ratio (OR) and 95% confidence interval (CI) of disease severity and malnutrition for food group intakes within 2 adjusted models. In a comparison of the highest with the lowest median of food groups intake, boiled potato (model 2, OR: 0.154, CI: 0.040-0.592) and legumes intake (model 2, OR: 0.090, CI: 0.020-0.406) significantly reduced 

risk of malnutrition, while, any consumption of mayonnaise increased malnutrition risk in both adjusted models. A decrease in disease severity was observed with increased consumption of nuts (model 2, OR:0.140, CI:0.031-0.625) and olive (model 2, OR:0.212, CI:0.049-0.917), whereas consumption of solid fats (model 1, OR:3.324, CI:1.080-10.238) was associated with increased risk of disease severity in model 1.

## Discussion

In the present study, we assessed the relation between food group’s intake and severity of hepatic cirrhosis and malnutrition status. Although limited studies have shown the role of nutrition in liver disease prognosis (11-15), the role of various food groups intakes regarding disease severity and malnutrition remains open to debate.

Thirteen major dietary groups were analyzed and assessed in this study. Intakes of nuts and olive inversely, and solid fats positively associated with severity of disease, while boiled potatoes and legumes consumption were associated with lower risk, and mayonnaise was associated with higher risk of malnutrition in these cirrhotic patients. Although there is no study assessing the association between food groups intake and Child-Pugh score and malnutrition status, few previous studies have reported some evidence which was in accordance with our findings.

A study by Soto-alarcon *et al.* reported several protective effects of extra virgin olive oil on the liver, like reducing hepatic steatosis, hepatocyte ballooning, fibrogenesis and preventing lipid oxidation. In addition, extra virgin olive oil prevented inflammation, oxidative stress, endoplasmic reticulum stress, mitochondrial dysfunction and insulin resistance through inactivation of the nuclear transcription factor-κB (NF- κB) and inhibition of the protein kinase RNA-like endoplasmic reticulum kinase (PERK) pathways. These effects might be due to high level of oleic acid and phenolic compounds like hydroxytyrosol and oleoropein, which have anti-oxidants properties (16).

Nuts are nutritionally dense fruits, consisting of a unique blend of essential nutrients, fatty acids, and bioactive compounds. In a recent work at the Storr Liver Unit, tree nuts and walnuts in particular, improved liver function tests in patients with NAFLD through improvements of inflammation, lipid profile and hepatic steatosis (17). 

In our study, legumes consumption was associated with lower risk of malnutrition. Jenkins *et al.* suggested that higher intake of vegetable protein like legumes improve carbohydrate tolerance in cirrhotic patients. Lentil in comparison with bread and cottage cheese as breakfast decreased blood glucose and insulin due to its high content of fiber (18). 

Weber *et al.* compared the effects of a vegetable protein versus animal protein to find the components that have therapeutic effects in hepatic encephalopathy. Vegetable diet might exert beneficial effects on nitrogen balance. The key difference between animal and vegetable protein is in their amino acid profiles. Therapeutic effect of vegetable protein can be explained by reduced amount of methionine and aromatic amino acids. Moreover, high amount of fiber and complex polysaccharides, improve bacteria metabolism and increase nitrogen excretion into fecal bacteria (19).

Moreover, Bianchi *et al.* compared the effects of mainly vegetable protein diet with an animal protein diet in cirrhotic patients and chronic permanent encephalopathy. They identified better nitrogen balance during the vegetable protein diet because of reduced urinary nitrogen excretion. In addition, plasma amino acids, ammonia, insulin, and clinical grading of encephalopathy were lower in vegetable protein diet compared with animal diet (20).

Our study had some limitations including selection bias, which should also be considered in interpreting the results. In addition, we used SGA for assessment of malnutrition, which is a subjective measurement. Another problem was low number of Child-Pugh C patients, which ultimately leaves the sample inhomogeneous. This fact may jeopardize the results of the associations with the staging of the disease. Eventually, we could not directly infer causality due to the cross-sectional nature of the study design. 

The strengths of this study were high participation rate, using valid questionnaires and lack of residual confounding (by adjustment for important confounders). In addition, this is the first study which examined the relation between food groups intake and severity of disease and nutritional status in patients with cirrhosis. 

In conclusion, our findings suggested the protective effect of healthy foods such as nuts and olive on severity of hepatic cirrhosis (Child-Pugh score), and boiled potato and legumes on malnutrition status (SGA). Conversely, unhealthy food selection including solid fats and mayonnaise group were positively associated with Child-Pugh score and malnutrition respectively. Since we have a small number of patients, it is suggested to carry out new studies with prospective design to prove the present results.
